# The Effect of TiC–TiB_2_ Dual-Phase Nanoparticles on the Microstructure and Mechanical Properties of Cast Ni–Fe-Based Superalloys

**DOI:** 10.3390/ma17235781

**Published:** 2024-11-25

**Authors:** Guanlan Liu, Shengwei Sun, Yaoyun Hu, Qinglong Zhao

**Affiliations:** 1 Key Laboratory of Automobile Materials, Ministry of Education and School of Materials Science and Engineering, Jilin University, No. 5988 Renmin Street, Changchun 130022, China; liuguanlan5651@163.com (G.L.); sswstudy_l@163.com (S.S.); 2 FAW JieFang Automotive Co., Ltd., Changchun 130011, China; 3 China Energy Engineering Group Tianjin Electric Power Design Co., Ltd., Tianjin 300400, China; hyaoyun@163.com

**Keywords:** Ni–Fe-based superalloys, TiC–TiB_2_ nanoparticles, γ’ precipitates, mechanical properties

## Abstract

TiC–TiB_2_ dual-phase nanoparticles were added into a Ni–Fe-based cast superalloy and their effects on the microstructure and mechanical properties were compared to those of a Ni–Fe-based superalloy with the addition of TiC nanoparticles. The addition of TiC nanoparticles led to the precipitation of a higher volume fraction of carbides. Compared to the addition of TiC, the addition of TiC–TiB_2_ nanoparticles not only led to the precipitation of carbides but also promoted the formation of flaky borides and a reduction in the precipitation of the Laves phase. The strengthening effect of TiC–TiB_2_ nanoparticles on the mechanical properties of Ni–Fe-based superalloys was stronger than that of TiC nanoparticles due to more secondary γ’ precipitates. This study provides valuable insights for selecting ceramic nanoparticles to increase the mechanical properties of cast Ni–Fe-based superalloys.

## 1. Introduction

Casting superalloys are widely used in aviation, aerospace, and energy industries due to their low production cost, simple manufacturing process, and excellent mechanical properties [1,2,3,4]. The rapid development of modern industry requires further improvement in the microstructure and mechanical properties of superalloys [5,6,7].

In recent decades, ceramic nanoparticles have been widely used as reinforcements in metal-based composites. TiC nanoparticles are widely used to effectively improve the mechanical properties of Ni-based superalloys due to the grain refinement and the efficient hindrance on the mobility of dislocations [8,9,10,11]. Liu et al. [12] added trace TiC nanoparticles to the cast K214 superalloys, which promoted grain refinement, decreased the segregation of alloying elements, and enhanced the strength and elongation. Hu et al. [10] prepared in situ TiC-reinforced nickel matrix composites by using the Ti_2_AC intermediate alloy. The experiment demonstrated the semi-coherence of the interface between TiC and Ni matrix, and the orientation relationship was (020)_TiC_//(1(_)1(_)1(_))_Ni_ and [001]_TiC_//[011]_Ni_. The fabricated composites exhibit substantially higher yield strengths of 654 ± 38 MPa and 769 ± 44 MPa than monolithic Ni. Wei et al. [13] successfully prepared TiC-reinforced René 104 superalloy by SLM with micron-sized TiC particles. The TiC nanoparticles provided heterogeneous nucleation, which effectively promoted grain refinement and columnar-to-equiaxed grain transition. The cracking density was reduced by 83.3% due to the formation of fine equiaxed grains. Wang et al. [14] prepared a very dense TiC/GTD222 composite with satisfactory SLM processing properties in a wide processing window. The nano-size carbide was distributed at the cell boundaries in the SLMed TiC/GTD222 composite, and the interface between the carbide and the matrix was incoherent. The yield strength of the SLMed TiC/GTD222 composite was 289 MPa higher than that of the SLMed GTD222 alloy due to multiple strengthening mechanisms, including Orowan strengthening, load transfer, Hall-Petch effect, and dislocation strengthening. Hong et al. [15] produced ultrafine TiC particle-reinforced Inconel 625 composite parts by laser metal deposition additive manufacturing process, and TiC nanoparticles effectively refined the grains and improved the strength and elongation due to the efficient prohibition of ultrafine reinforcements on the mobility of dislocations. TiB_2_ is also widely used as reinforcement in superalloys. Zhang et al. [16] successfully fabricated Inconel 625/nano-TiB_2_ composites with excellent mechanical properties by laser-aided additive manufacturing process. The TiB_2_ nanoparticles reinforced the grain boundary and significantly increased the material strength. The strengthening and toughening efficiency of TiC–TiB_2_ dual-phase nanoparticles on aluminum alloys is stronger than that of TiC single-phase nanoparticles, which has also been confirmed in aluminum and magnesium alloys [17,18]. However, the effect of TiC–TiB_2_ dual-phase nanoparticles on the microstructure and mechanical properties of Ni-based superalloys has not been explored.

In this work, a Ni–Fe-based superalloy with 0.5 wt.% TiC nanoparticles or TiC–TiB_2_ nanoparticles was fabricated by vacuum induction melting. The improvement of mechanical properties as well as the strengthening mechanisms were discussed. Meanwhile, the effects of TiC and TiC–TiB_2_ nanoparticles on the microstructure and mechanical properties of the Ni–Fe-based superalloys were investigated.

## 2. Materials and Methods

**Figure 1:**
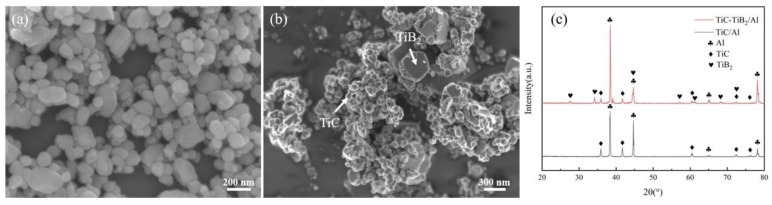
The morphology of (**a**) TiC nanoparticles and (**b**) TiC–TiB_2_ nanoparticles extracted from the intermediate alloys. (**c**) The XRD patterns of TiC/Al and TiC–TiB_2_/Al intermediate alloys.

The TiC/Al intermediate alloy was prepared by reaction sintering of Al:Ti:CNT (carbon nanotube) = 35:12:3 (mass ratio, C/Ti molar ratio of 1:1) powder mixture under high vacuum conditions. The TiC–TiB_2_/Al intermediate alloys were prepared using a powder mixture of Al:Ti:B_4_C = 8.4:2.6:1 (mass ratio) in the same conditions. The morphology of nanoparticles was characterized by scanning electron microscopy (JEOL Nippon Electronics Corporation, Tokyo Metropolis, Japan, JSM-6700F). The average diameter of TiC and TiB_2_ particles was ~102.5 nm and ~280.5 nm, respectively. The morphology of the TiC nanoparticles and TiC–TiB_2_ nanoparticles was spherical and prismatic, as shown in Figure 1a,b. Figure 1c shows the XRD pattern of the TiC/Al and TiC–TiB_2_/Al intermediate alloys.

The base alloy was an Al-modified Ni–Fe superalloy similar to Inconel 718Plus. The 9 g intermediate alloys were added to 1500 g melted Ni–Fe-based superalloys in an argon-protected vacuum induction furnace(Shenyang Hotstar New Materials Preparation Technology, Shenyang, China, HISM-5KG). The melt was held at 1500 °C for 10 min and then poured into a graphite mold. The additional amount of 0.5 wt.% TiC or TiC–TiB_2_ nanoparticles was added into the Ni–Fe-based superalloys, which were denoted as S1 and S2, respectively. The Ni–Fe-based superalloys without nanoparticles were denoted as S0. The alloy composition is shown in Table 1.

The samples were subjected to homogenization + solution + two-step aging heat treatment (SHT). Homogenization heat treatment was carried out at 1095 °C for 1 h to dissolve the δ phase and Laves phase and reduce composition segregation. The solution treatment was carried out at 955 °C for 1 h to further dissolve the δ phase and Laves phase and reduce the composition segregation. The two-step aging was successively held at 720 °C and 620 °C for 8 h, and the strengthening phase was precipitated to improve the mechanical properties of the superalloys [19].

The microstructure of superalloys was characterized by scanning electron microscopy (SEM, Phenom World, Eindhoven, The Kingdom of the Netherlands, Phenom Prox, and JEOL Nippon Electronics Corporation, Tokyo Metropolis, Japan, JSM-7900F). The samples for SEM characterization were mechanically ground, polished, and etched in the mixed reagent (92 mL HCl + 5 mL H_2_SO_4_ + 3 mL HNO_3_). NanoMeasurer V1.2 and Image J2 software were used to measure the size and area fraction of the second phase in the interdendritic region, respectively. At least 20 secondary electron images were measured for each sample. The gauge length of the high-temperature tensile specimen was 10 mm, and the cross-section size was 5.0 × 1.5 mm^2^. High-temperature tensile tests were performed using an electronic creep testing machine (RDL 50) at 650 °C at a tensile rate of 0.5 mm/min. At least three tests were repeated for each sample.

## 3. Results and Discussion

**Figure 2:**
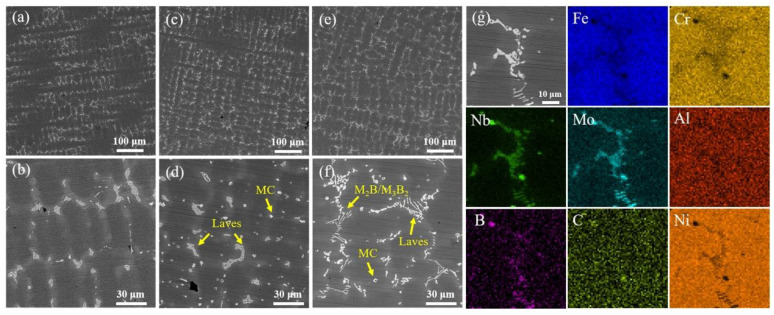
Microstructure of the as-cast (**a**,**b**) S0, (**c**,**d**) S1, and (**e**,**f**) S2 samples. (**g**) EDS analysis of boride and Laves phase in as-cast S2 sample.

The interdendritic region of Ni–Fe-based superalloys is generally composed of the Laves phase, MC carbides, borides, and γ’ precipitates. The area fractions of the second phases in the interdendritic regions of the as-cast S0, S1, and S2 samples are 3.42%, 3.58%, and 4.99%, respectively, as shown in Figure 2a,c,e. The island Laves phase with a size of 8–10 μm is enriched in the interdendritic region of the S1 sample, and a large number of MC carbides are precipitated due to the introduction of TiC nanoparticles, as shown in Figure 2d. The number density of the Laves phase in the interdendritic region of the S2 sample is reduced, and the morphology is changed from island to skeleton or block. A small amount of MC carbides is also precipitated in the interdendritic regions of the S2 sample, as shown in Figure 2f. A large number of lamellar eutectic borides were precipitated near the Laves phase in the S2 sample. The types of borides usually include M_2_B and M_3_B_2_ [20,21,22,23]. According to the EDS results, it is found that Mo and Cr elements are enriched in these lamellar borides, as shown in Figure 2g [24].

**Figure 3:**
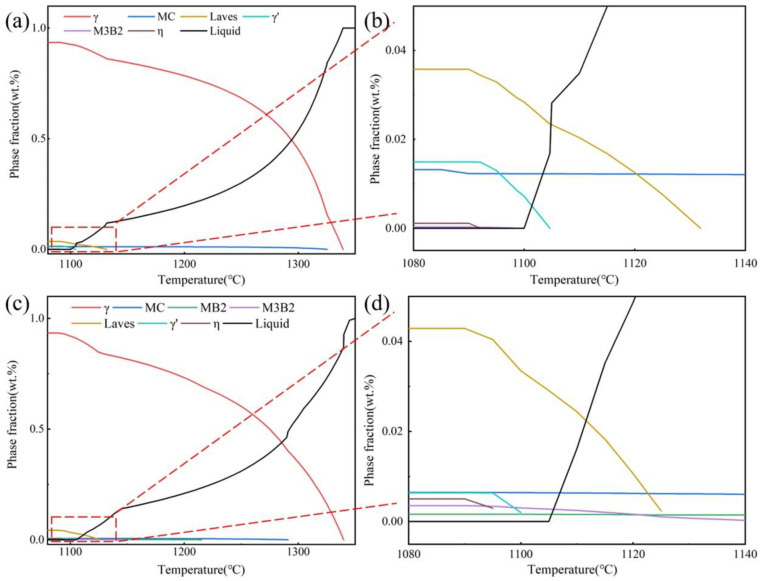
The phase fraction of the (**a**,**b**) S1 sample and (**c**,**d**) S2 sample calculated by Jmatpro software. Figures (**b**) and (**d**) are the enlarged graphs in the red boxes of Figures (**a**) and (**c**) respectively.

The difference between the precipitates of the S1 sample and the S2 sample mainly lies in the type and content of carbides and borides. The added particles possibly dissolved, leading to an increase in C and B contents. The phase fraction (weight fraction) of S1 and S2 samples after solidification was calculated by Jmatpro Version 7.0 (database version is also 7.0) software. The basic model of phase transition calculation in the solidification process is the Scheil–Gulliver model. Figure 3 is the phase composition of the S1 sample and S2 sample calculated by Jmatpro software (v. 15.0). The main phase consists of γ, MC, MB_2_, M_3_B_2_, γ’, and η. The content of MC carbides in the S1 sample is significantly higher than that in the S2 sample, as shown in Figure 3a. A large number of M_2_B borides and M_3_B_2_ borides are precipitated in the S2 sample, as shown in Figure 3b.

**Figure 4:**
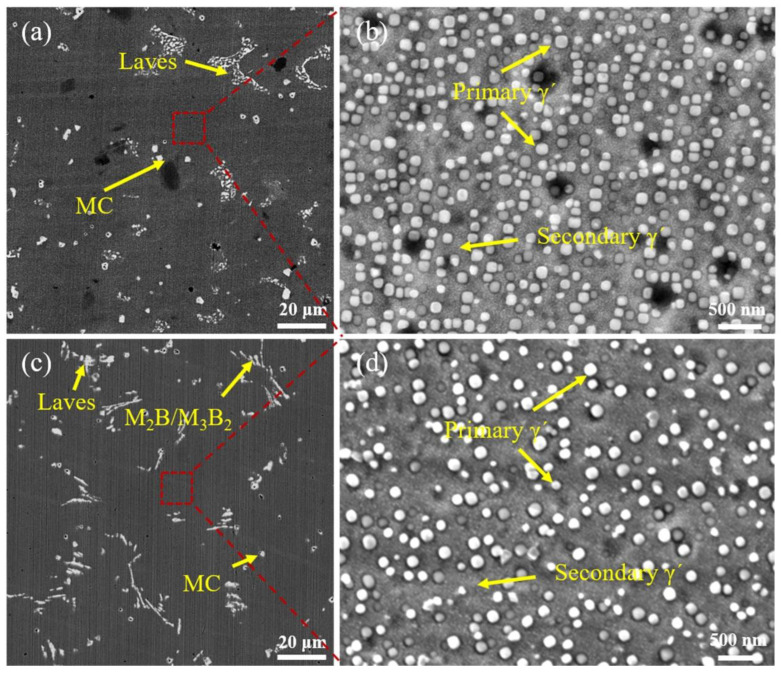
Microstructure of the (**a**,**b**) S1 sample and (**c**,**d**) S2 sample after SHT. Figures (**b**) and (**d**) are the enlarged graphs in the red boxes of Figures (**a**) and (**c**) respectively.

After SHT, the area fraction of the second phases in the interdendritic region in the S1 sample and S2 sample decreased to 2.32% and 1.87%, respectively, as shown in Figure 4a,c. According to Yan et al.’s research [25], lamellar borides dissolve rapidly during solution treatment compared with carbides. Therefore, the microsegregation of the S2 sample is reduced (4.99%→1.87%) during the SHT process. The Nb element released by the dissolution of the Laves phase is beneficial to the precipitation of γ’ phase [26]. The coarser primary γ’ and refined secondary γ’ strengthening phases are precipitated from the γ matrix [27,28]. The size of the uniformly distributed primary γ’ phase precipitated in the S1 sample is 80–120 nm. Compared with the S1 sample, the size of the primary γ’ strengthening phase precipitated in the S2 sample is 60–110 nm. The number density of primary γ’ precipitates in the S1 sample is larger than that in the S2 sample, as shown in Figure 4d. The primary γ’ and secondary γ’ precipitates are essentially composed of the same elements, so an increase in the content of the primary γ’ in the S1 sample leads to a decrease in the content of secondary γ’ precipitates.

**Figure 5:**
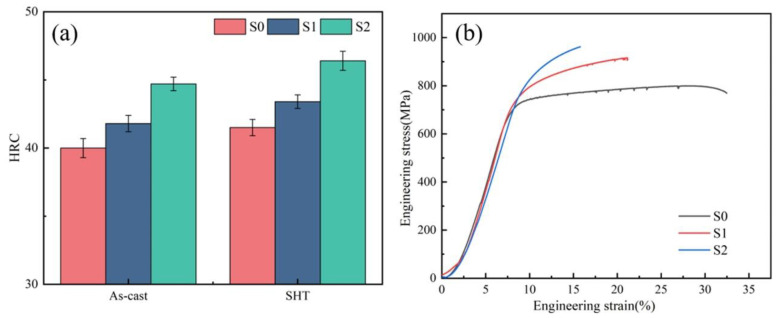
(**a**) The Rockwell hardness of S0, S1, and S2 samples. (**b**) The engineering stress-strain curves of the S0, S1, and S2 samples.

The Rockwell hardness of S0, S1, and S2 samples was tested, as shown in Figure 5a. The Rockwell hardness of the as-cast S0, S1, and S2 samples is 40 HRC, 41.8 HRC, and 44.7 HRC, respectively. After SHT, the Rockwell hardness of the S0, S1, and S2 samples is 41.5 HRC, 43.4 HRC, and 46.4 HRC, respectively. The increase in hardness of the S2 sample comes from a higher fraction of secondary γ’. Usually, the superalloys are used in the heat treatment state. Figure 5b shows the high-temperature tensile of the S0, S1, and S2 samples after SHT. The yield strength (YS), ultimate tensile strength (UTS), and elongation (EL) of the S0 sample are 659 MPa, 799 MPa, and 32.4%. The YS and UTS of the S2 sample are 770.7 MPa and 962 MPa, which are 11.7% and 5% higher than those of the S1 sample (YS = 690.2 MPa, UTS = 916 MPa). The EL of the S1 sample is 20.3%, which is higher than that of the S2 sample (EL = 14.6%). The unusual shape in the tensile curve is due to the error caused by the lack of an extensometer in the high-temperature tensile equipment.

The grain size of the cast S0, S1, and S2 samples was very coarse, in the order of 1 mm. The literature has reported that the effect of grain boundaries on strengthening is negligible when the grain size is larger than 500 μm [29,30]. Hosseini et al. have similar reports [31,32]. The borides in the Ni–Fe-based superalloys increase with the increase of the content of the B element, which is beneficial for the strength and plasticity of the Ni–Fe-based superalloys [33]. However, too many borides are harmful to elongation when the content of the B element is too high. The B content in the S2 sample is 0.08 wt.%. After SHT, more flaky borides are precipitated in the S2 sample, and the borides with a large diameter-to-thickness ratio have a stronger load transfer strengthening effect than carbides [34].

Al and Ti elements are the main components of γ’ precipitates, and increasing the content of Al and Ti elements is beneficial to the precipitation of γ’ phase [35]. Similar to Inconel 718Plus superalloys, the main strengthening phases of S1 and S2 samples consist of primary and secondary nanoscale L_12_-type γ’ -Ni_3_(Al, Ti, Nb) phases uniformly distributed on the γ matrix, which enables high operating temperatures to be achieved [36]. The S2 sample contains the primary γ’ phase with a size of 100 nm and a smaller secondary γ’ phase with a size of less than 20 nm. Therefore, it can be concluded that the dominant mechanism of dislocation passage is to cut through the secondary γ’ phase and bypass the primary γ’ phase by creating Orowan loops around them. The strong pair coupling effect of the high-energy antiphase boundary (APB) mechanism explains the strengthening effect of the secondary γ’ precipitates. The addition of B element hinders the formation of primary γ’ phase so that more secondary γ’ phase can be formed. The average size of the γ’ phase (primary + secondary) is reduced, which means that the movement of dislocations is more hindered. In addition, the higher the amount of secondary γ’ phase, the greater the contribution of the shearing mechanism to precipitation strengthening [27,37,38]. Coarser γ’ precipitates increase both the dwell crack growth resistance and the elongation-to-failure [39]. The size of the secondary γ’ precipitate is 10 ~ 36 nm. The finely distributed secondary γ’ precipitates significantly hinder dislocation slip and enhance the tensile yield stress [27]. Therefore, TiC–TiB_2_ nanoparticles have a stronger strengthening effect than TiC nanoparticles but cause a reduction in elongation.

**Figure 6:**
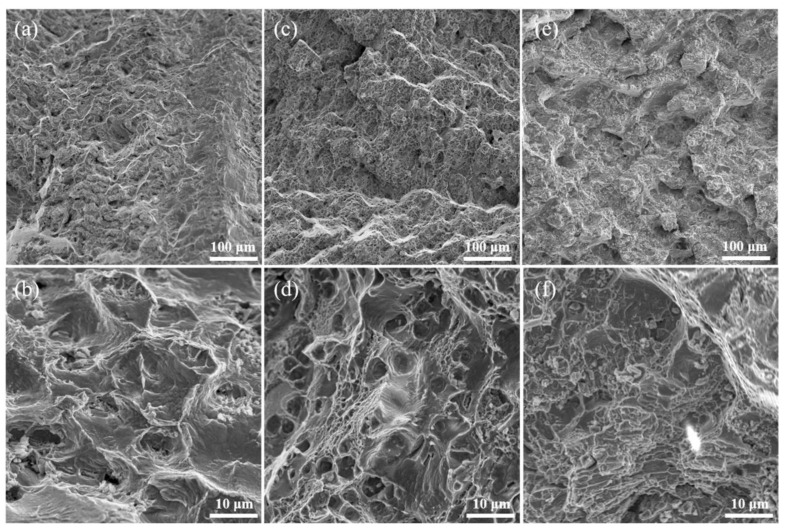
The fracture morphology of the (**a**,**b**) S0; (**c**,**d**) S1, and (**e**,**f**) S2 samples in SHT state after high-temperature tensile.

The fracture morphology of the samples in SHT state after high-temperature tensile was analyzed. The fracture morphology of the S0 sample is mainly fine dimples, as shown in Figure 6a,b. The larger dimples are dominant in the S1 sample, as shown in Figure 6c,d. The cleavage plane and micropores caused by the large-sized second phase are dominant in the S2 sample, indicating that the Ni–Fe-based superalloys are transformed into cleavage fractures. The S2 sample has lower plasticity than the S0 sample and S1 sample, as shown in Figure 6e,f.

## 4. Conclusions

1. After adding the same content of TiC–TiB_2_ and TiC nanoparticles, the added particles possibly dissolved, leading to an increase in C and B contents. A large number of MC carbides are precipitated by adding TiC nanoparticles. The addition of TiC–TiB_2_ nanoparticles makes a part Laves phase morphology change from island to skeleton or block, and many flaky borides are precipitated.
2. Jmatpro was used to calculate the phase composition of the S1 sample and S2 sample after solidification. The content of MC carbides in the S1 sample is significantly higher than that in the S2 sample. The borides precipitated in the S2 sample mainly consist of M_2_B and M_3_B_2_, and the content of carbides precipitated is less than that of the S1 sample.
3. After the SHT process, the primary γ’ precipitates in the Ni–Fe-based superalloys with TiC–TiB_2_ nanoparticles are smaller, which is beneficial to improve the strength due to the higher critical cut-through stress. The secondary γ’ precipitates that effectively hinder dislocation slip have a larger number density in the Ni–Fe-based superalloys with TiC–TiB_2_ nanoparticles. The YS and UTS of the Ni–Fe-based superalloys with TiC–TiB_2_ nanoparticles are higher than those with TiC nanoparticles, but the elongation is reduced.

## Figures and Tables

**Table 1:** The chemical composition of cast samples (in wt.%).

Sample	C	B	Mo	Al	Ti	Nb	Cr	Fe	Ni
S0	0.054	0.0035	3.03	0.61	1.56	4.91	17.36	19.66	Bal.
S1	0.16	0.0031	2.98	1.81	1.99	4.83	17.24	19.33	Bal.
S2	0.076	0.080	3.00	1.81	1.87	4.85	17.14	19.40	Bal.

## Data Availability

The data presented in this study are available on request from the corresponding author due to ongoing study.
